# Study on Material Removal Model by Reciprocating Magnetorheological Polishing

**DOI:** 10.3390/mi12040413

**Published:** 2021-04-08

**Authors:** Rensheng Wang, Shichao Xiu, Cong Sun, Shanshan Li, Xiangna Kong

**Affiliations:** 1 School of Mechanical Engineering, Liaoning Institute of Science and Technology, Benxi 117004, China; realwrs@163.com; 2 School of Mechanical Engineering and Automation, Northeastern University, Shenyang 110819, China; sgdlss33@163.com (S.L.); amyinchina@yeah.net (X.K.)

**Keywords:** reciprocating magnetorheological polishing (RMRP), material removal model, simulation analysis, polishing experiments, material removal rate (MRR)

## Abstract

In this study, a new reciprocating magnetorheological polishing (RMRP) method for a flat workpiece was proposed. Based on the RMRP principle and Preston equation, the material removal rate (MRR) model of the RMRP as well as its normal polishing pressure model was established. On this basis, the effects of different technological parameters including workpiece rotation speed, eccentric wheel rotation speed and eccentricity on the MRR of the workpiece were investigated. The K9 optical flat glass was polished with the RMRP setup to verify the MRR model. The experimental results showed that the effect of workpiece rotation speed on the MRR was much greater than that of eccentric wheel rotation speed and eccentricity, and the MRR increased from 0.0115 ± 0.0012 to 0.0443 ± 0.0015 μm/min as workpiece rotation speed rose. The optimum surface roughness reduced to Ra 50.8 ± 1.2 from initial Ra 330.3 ± 1.6 nm when the technical parameters of the workpiece rotation speed of 300 rpm, the eccentric wheel rotation speed of 20 rpm and the eccentricity of 0.02 m were applied. The average relative errors between the theoretical and experimental values were 16.77%, 10.59% and 7.38%, respectively, according to the effects of workpiece rotation speed, eccentric wheel rotation speed and eccentricity on MRR.

## 1. Introduction

With the continuous development of optoelectronic technique, the application of optical components plays a very important role for precision optics, defense technology, the aerospace industry, the microelectronics industry and medical equipment [1,2]. Typically, the processing requirements of the optical workpiece are relatively strict, for example, high surface accuracy, high-quality surface and less subsurface damage layer, etc. To date, numerous technologies have been developed for processing optical components such as bonnet tool polishing (BTP) [3], elastic emission machine (EEM) [4], ion beam figuring (IBF) [5], magnetorheological jet polishing (MJP) [6], chemical mechanical polishing (CMP) [7] and magnetorheological polishing (MRP) [8], etc. MJP is not only inefficient in processing, and the subsurface of the workpiece is damaged. BTP can achieve higher processing efficiency, but the subsurface of the workpiece may be damaged. Although the damage-free subsurface can be obtained by EEM and IBF, the processing efficiency of them is limited. CMP has done a good job in processing efficiency and surface quality, but the environment is affected during processing. In short, the aforementioned manufacturing technologies can polish an optical workpiece; however, the disadvantages are still obvious. MRP is considered the “gentle” manufacturing technology, and the shortcomings in the above processing can be effectively compensated [9,10,11,12].

Magnetorheological polishing (MRP) is a new ultra-precision machining technology proposed by Kordonski and his collaborators in the 1990s of the 20th century, which uses the magnetorheological effect (MR effect) to finish the optical workpiece surface. Its advantages can improve the machining accuracy and quality of a complex shape workpiece under the condition of high efficiency and it being non-destructive [13,14,15,16]. During the polishing process, the magnetic particles in the magnetorheological polishing fluids (MRP fluids) are magnetized to form a chain columnar magnetic structure under the magnetic field, which is consistent with the direction of the magnetic line of induction. In the meantime, the abrasive particles clamped in the magnetic chain are affected by the clamping force. Based on magnetohydrodynamics theory, the magnetic particles in MRP fluids are attracted to the strong magnetic area by the magnetic field. The abrasive particles in MRP fluids are pushed to the weak magnetic area with contacting the workpiece surface by the buoyancy of the magnetic fluids. Therefore, an obvious shear yield stress is generated in the system, and the ultra-precision machining technology of deterministic polishing is completed [17,18,19]. It was reported that complex optics with a figure accuracy < 50 nm and a surface roughness < Ra 1 nm were produced by MRP [20]. Afterwards, some new MRP technologies had been derived from it. Kim et al. [21] proposed a surface-finishing method for three-dimensional microchannel structures using magnetorheological fluids. When a microchannel was polished by Kim’s method, the roughness of bottom and side surfaces of the silicon channel was reduced by 5–10 times. Seok et al. [22] developed a fabrication method of curved surfaces on silicon-based micro-structures using magnetorheological finishing. After 2 h of polishing time, the surface roughness value of the workpiece was reduced from Ra 420.62 to Ra 38.1 nm by the proposed method. Singh et al. [23] designed a new precision finishing process for the nanofinishing of 3D surfaces using a ball end magnetorheological finishing tool. The polishing head mainly comprises a central rotating inner core, a stationary electromagnet coil and a stationary outer core. The MRP fluids can be delivered from the upper end of the polishing head to the tip of the polishing head along the MRP fluids flow passage in the central rotating inner core. On the other hand, the magnetic field flows through inner core to outer core along the tip of the polishing head. Thus, the MRP fluids form a spherical polishing tool at the tip of the polishing head under the magnetic field similar to the ball end mills for polishing freeform surfaces [24]. The groove surface roughness value of the non-ferromagnetic workpiece was reduced from Ra 336.8 to Ra 102 nm with a polishing time of 60 min using ball end magnetorheological finishing.

As a measure of the machining results of magnetorheological polishing, the material removal rate (MRR) of the workpiece surface had been studied by many scholars. Although the MRR mathematical model was established from different perspectives because of multiple influence factors during the MRP process, most of the MRR mathematical models were predicted based on the Preston equation. Shorey [25] found the MRR was proportional to the drag force by measuring drag force on a sapphire and proposed a modified Preston coefficient for the MRR mathematical model. DeGroote et al. [26] put forward an improved model for the MRR of MRP by incorporating conventional CMP and MRP. The improved model depicted that peak removal rate was proportional to the five terms. Miao et al. [27] presented a modified Preston equation, which reasonably estimated the MRR for the workpiece (optical glasses) with incorporating mechanical properties, shear stress, and velocity. Furthermore, Li et al. [28] established the MRR for a shear thickening polishing (STP) based on the Preston equation, fluid dynamics and shear thickening mechanism. Wang et al. [29] constructed the MRR model by using the tangential force and normal force on the workpiece and concluded that the removal effect during the MRP process was more dominant by the tangential force on the workpiece.

As stated above, despite the numerous MRR model predictions for MRP, most MRR models were suitable for the material removal of a non-flat workpiece, and the MRR model of a flat workpiece as well as its normal polishing pressure model was rarely established. To solve this problem, in this paper the material removal principle was studied for a flat workpiece based on the reciprocating magnetorheological polishing (RMRP) method, and the MRR model as well as the normal polishing pressure model was established by the RMRP. Moreover, the effects of various technological parameters on the MRR of the workpiece were investigated theoretically and experimentally. Furthermore, the correctness of the MRR model of RMRP was validated.

## 2. MRR Model of the RMRP

### 2.1. Material Removal Principle of the RMRP

**Figure 1:**
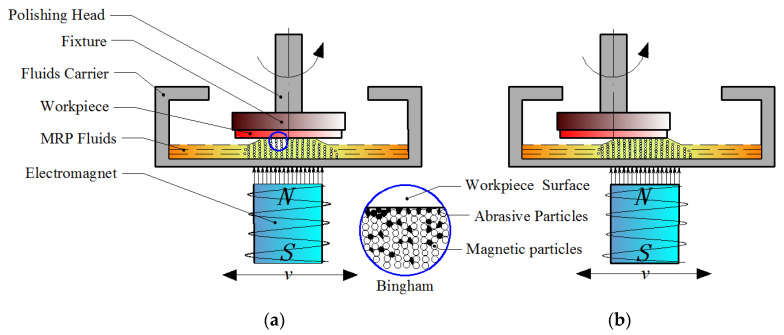
Principle diagram of the reciprocating magnetorheological polishing (RMRP): (**a**) the processing area at the center area of the workpiece; (**b**) the processing area at the edge area of the workpiece.

**Figure 2:**
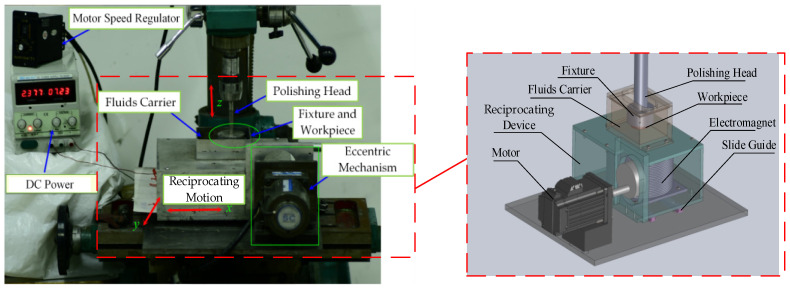
Experimental setup of the RMRP.

The RMRP is a new machining method that combines the two movements of magnetic field translation and workpiece rotation to polish the flat workpiece. The electromagnet and the fluid carrier as integrated structures are driven by the reciprocating device, and the workpiece clamped in the fixture is driven to rotate around the axis by the polishing head. The principle diagram of RMRP is as given in Figure 1. According to the principle of the RMRP, due to the continuous change of the position of magnetic field, the position of the flexible polishing brush formed by the MR effect of the MRP fluids changes accordingly. When the electromagnet moves to the center area of the workpiece, the polishing brush with a certain hardness and elasticity is formed by the MRP fluids corresponding to this area in the fluid carrier, and it becomes a polishing tool that can undertake larger shear force and control polishing force, as shown in Figure 1a. During the polishing process, the polishing debris is deposited on the bottom of the fluid carrier away from the processing area, which is conducive to MRP fluid renewal in the processing area. When the electromagnet moves radially along the workpiece to its edge area, the formed polishing tool also moves there, and at this time the edge area of the workpiece is polished, as shown in Figure 1b. Because of the workpiece only rotating in a fixed position, the magnetic polishing brush moves continuously with the electromagnet, which realizes that the electromagnet reciprocates for one cycle every time, and the workpiece surface is polished once in a full range with polishing tools [30]. Based on the principle shown in Figure 1 and the coverage of the magnetic line to the workpiece (the polishing brush was in fully contact and friction with the workpiece), the experimental setup used for the RMRP was developed as shown in Figure 2.

In order to ensure the machining accuracy of the workpiece, the experimental setup errors cannot be ignored. The main sources of errors are the machining errors of the polishing head (the perpendicularity error between the shaft center of the polishing head and the upper surface of the fixture) and the installation errors of the workpiece (the clamping error between the fixture and the workpiece), which cause the errors of working gap (the parallelism error between the machined surface of the workpiece and the inner bottom face of the fluid carrier). In order to reduce the machining errors of the workpiece, the verticality error of the experimental setup is controlled within 0.015 mm and the clamping error can be adjusted by the installation of the workpiece, then the working gap error is less than 0.03 mm. When the working gap is set to 1 mm, the parallelism error is equivalent to 3% of the machining gap, which is acceptable.

### 2.2. MRR Model of the RMRP

The MRR model of the RMRP was established in this section based on the material removal principle of the RMRP. During the RMRP process, because the surface of the workpiece and the inner bottom face of the fluid carrier were both planar structures, there was not fluid hydrodynamic pressure, and the polishing pressure acting on the workpiece was only magnetization pressure that was normal pressure on the workpiece surface. Based on the Preston equation, as shown in Equation (1), the material removal mechanism of the MRP was regarded as the construction of a certain pressure field and relative speed by external conditions. The microscopically rough surface was squeezed, cut or ploughed with using the micro-blade of the polishing tool formed by polishing particles.

(1) MRR=kpv

where k is the Preston coefficient that is a constant, p is the pressure of the flexible polishing brush on the workpiece surface, and v is the relative speed between the workpiece surface and the abrasive particles of MRP fluids.

Since the polishing brush was reciprocating linear motion in the polishing process, the polishing pressure acting on the workpiece was constantly changing with the movement of the polishing brush, and the polishing pressure of each area on the workpiece was equal. In other words, the probability of the polishing brush acting on each polishing area of the workpiece during the polishing process was equal based on the MR effect. Therefore, the viscoelastic cylindrical polishing brush is formed by the MRP fluids under the magnetic field, and the polishing pressure of RMRP can be expressed by a certain value, which is given as follows [31]:

(2) $$P=-\frac{1}{K}\left(\frac{\Delta V}{V}\right)$$

where K represents the compressibility coefficient of the polishing brush, which is related to the concentration of MRP fluids, V represents the volume of the polishing brush, and ΔV represents the volume variation of the polishing brush.

The volume relation in Equation (2) can be expressed as:

(3) $$\frac{\Delta V}{V}=2\frac{\Delta R}{R}+\frac{\Delta h}{h}$$

where R is the radius of the polishing brush formed by the MR effect and h is the working gap. The polishing brush is a cylindrical axisymmetric structure, and the working gap is limited by initial conditions—that is, Δh/h=0.

From the relationship between the material strain and stress, the radial strain of the polishing brush can be expressed as: $\varepsilon_{r}=\Delta R/R=\sigma_{r}/E$. Thus, Equation (2) can be expressed as:

(4) $$P=-\frac{2\sigma_{r}}{KE}$$

where E and $\sigma_{r}$ represent Young’s modulus and the radial stress of the polishing brush, respectively.

**Figure 3:**
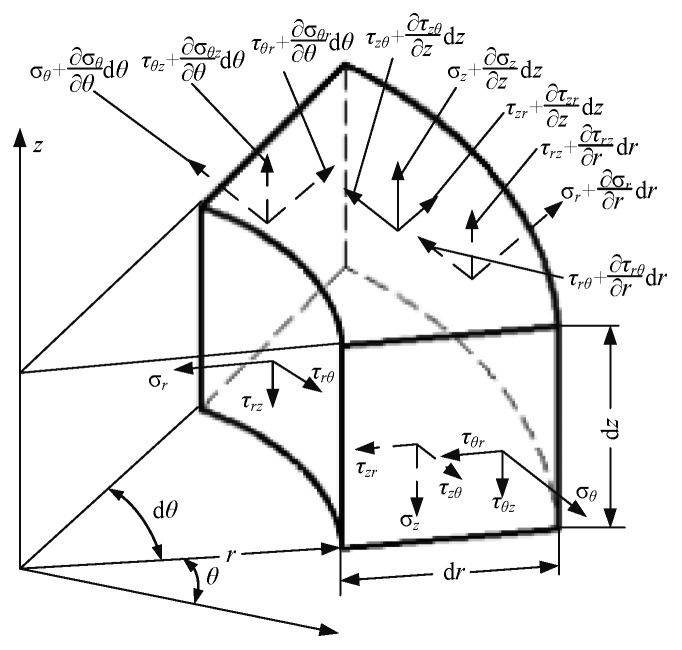
Stress model of an element block in the cylindrical coordinate system.

In accordance with the polishing brush being a cylindrical axisymmetric structure, the stress model of the element block can be established in the cylindrical coordinate system (r, θ, z), as shown in Figure 3.

The radial stress of the polishing brush is calculated, and the equilibrium differential equation is given as follows:

(5) $$\begin{array}{l} \frac{\partial \sigma_{r}}{\partial r}+\frac{1}{r}\frac{\partial \tau_{\theta r}}{\partial \theta }+\frac{\partial \tau_{zr}}{\partial z}+\frac{\sigma_{r}-\sigma_{\theta }}{r}+f_{r}=0\\ \frac{\partial \tau_{r\theta }}{\partial r}+\frac{1}{r}\frac{\partial \sigma_{\theta }}{\partial \theta }+\frac{\partial \tau_{z\theta }}{\partial z}+\frac{2\tau_{r\theta }}{r}+f_{\theta }=0\\ \frac{\partial \tau_{rz}}{\partial r}+\frac{1}{r}\frac{\partial \tau_{\theta z}}{\partial \theta }\frac{\partial \sigma_{z}}{\partial z}+\frac{\tau_{rz}}{r}+f_{z}=0 \end{array}$$

Therefore, the radial stress of the polishing brush can be expressed as follows:

(6) $$\sigma_{r}=-f_{r}\cdot r+C$$

where f_r_ is the radial volume force of the element block, which is composed of rotating centrifugal volume force and radial magnetic field volume force, r is the radial displacement of the element block, and C is the constant.

Thus,

(7) $$\sigma_{r}=\left(\rho \omega^{2}r-F_{r}\right)\cdot r+C$$

where ρ is the density of MRP fluids, ω is the angular velocity of the element block, and F_r_ is the radial magnetic field volume force.

According to the theory of electromagnetism, the radial magnetic field volume force can be expressed as follows [32]:

(8) $$F_{r}=\frac{\mu_{0}\left(\mu_{r}-1\right)}{2}\frac{\partial H_{r}^{2}}{\partial r}$$

where $\mu_{0}$, $\mu_{r}$ and $H_{r}$ represent the permeability of vacuum, the relative permeability of MRP fluids and the radial magnetic field intensity, respectively.

The radial magnetic field intensity $H_{r}$ of the element block can be solved by the fitting function as follows:

(9) $$\begin{array}{ll} H_{r}\left(h,r\right)= & -866.8+2.91\times 10^{6}r-4\times 10^{5}h-7.14\times 10^{7}r^{2}+\\ & 1.41\times 10^{7}h^{2}-8.2\times 10^{7}hr \end{array}$$

where h and r represent the working gap and the radial displacement of the element block, respectively.

The polishing pressure of RMRP can be solved by Equation (4) to Equation (9):

(10) $$P=\frac{1}{KE}\left[\mu_{0}\left(\mu_{r}-1\right)r\frac{\partial H_{r}^{2}}{\partial r}-2\rho \omega^{2}r^{2}\right]+C_{1}$$

By integrating the polishing pressure (Equation (10)) in the contact area during the MRP process, the total polishing pressure of the workpiece can be obtained, which can be expressed as:

(11) $$P^{\prime }=\int_{0}^{2\pi }d\theta \int_{0}^{R}\left\{\frac{1}{KE}\left[\mu_{0}\left(\mu_{r}-1\right)r\frac{\partial H_{r}^{2}}{\partial r}-2\rho \omega^{2}r^{2}\right]+C_{1}\right\}rdr$$

Afterwards, the total polishing pressure is divided by the contact area, which is the average polishing pressure and equals the normal pressure of the flexible polishing brush on the workpiece surface. The normal pressure p is calculated by the following equation:

(12) $$p=\frac{P^{\prime }}{\pi R_{1}^{2}}=\frac{1}{\pi R_{1}^{2}}\int_{0}^{2\pi }d\theta \int_{0}^{R}\left\{\frac{1}{KE}\left[\mu_{0}\left(\mu_{r}-1\right)r\frac{\partial H_{r}^{2}}{\partial r}-2\rho \omega^{2}r^{2}\right]+C_{1}\right\}rdr$$

where R and R_1_ separately refer to the radius of the polishing brush and workpiece, and C_1_ represents a constant.

**Figure 4:**
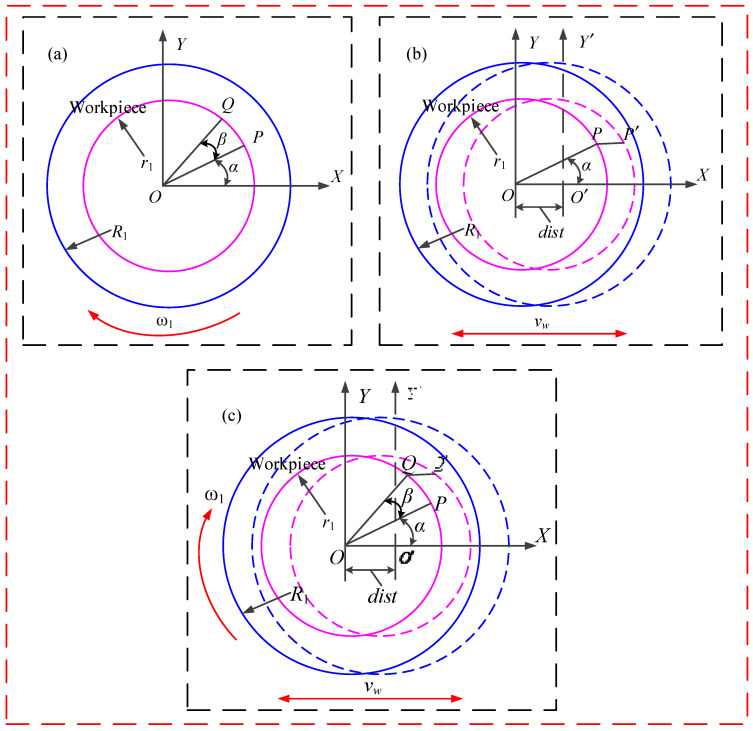
Kinematic diagram of the abrasive particle: (**a**) kinematic diagram of the abrasive particle during the rotation of the workpiece; (**b**) kinematic diagram of the abrasive particle during the translation of the workpiece; (**c**) kinematic diagram of the abrasive particle during the rotational and translational motion of the workpiece.

According to the Preston equation, in addition to the normal pressure on the workpiece surface, the relative speed between the workpiece surface and the abrasive particles of MRP fluids is also an important influencing factor of material removal rate. Figure 4 shows the kinematic diagram of the abrasive particle. When the workpiece only rotates in the experimental setup, assuming that the polishing abrasive particle starts at point P, the polishing abrasive particle ends at point Q after a period of polishing time of t, as shown in Figure 4a. Then, the trajectory model of an abrasive particle in the coordinate system XOY is shown in the following equation:

(13) $$\{\begin{array}{l} x=r_{1}\cos\left(\alpha +\beta \right)\\ y=r_{1}\sin\left(\alpha +\beta \right) \end{array}$$

where α is the initial angle of the abrasive particle, β is the rotation angle of the workpiece after a period of polishing time, β=ωt, and r_1_ is the distance between abrasive particle P point and workpiece center O point.

When the fluid carrier only translates in the experimental setup, assuming that the polishing abrasive particle similarly starts at point P, the polishing abrasive particle ends at point P′ after a period of polishing time of t, as shown in Figure 4b. Then, the trajectory model of the abrasive particle in the coordinate system XO′Y′ is shown in the following equation:

(14) $$\{\begin{array}{l} x^{\prime }=r_{1}\cos\alpha +\mathrm{dist}\\ y^{\prime }=r_{1}\sin\alpha \end{array}$$

where dist describes the coordinate position (m) of the abrasive particle’s reciprocating motion in the working stroke L, $\mathrm{dist}=v_{w}t$ ($v_{w}$ represents the reciprocating velocity of the fluid carrier) and dist∈[−L/2,L/2].

Because the reciprocating device is mainly composed of an eccentric wheel, the reciprocating velocity is controlled by the angular velocity and eccentricity of the eccentric wheel. The reciprocating velocity can be expressed as:

(15) $$v_{w}=e\omega_{0}\sin\omega_{0}t$$

where ω_0_ and e represent the angular velocity and eccentricity of the eccentric wheel, respectively.

During the RMRP, the motion trajectory of the polishing abrasive particle is formed by superimposing the two independent motion modes in the above-mentioned reciprocating polishing system, assuming that the polishing abrasive particle starts at point P and the polishing abrasive particle ends at point Q′ after a period of polishing time of t, as shown in Figure 4c. The trajectory model of the abrasive particle can be expressed as follows:

(16) $$\{\begin{array}{l} x^{\prime }=r_{1}\cos\left(\omega t+\alpha \right)+v_{w}t\\ y^{\prime }=r_{1}\sin\left(\omega t+\alpha \right) \end{array}$$

Based on Equation (16), the relative speed between the workpiece surface and the abrasive particles of MRP fluids can be derived as follows:

(17) $$\{\begin{array}{l} v_{x}=-\omega r_{1}\sin\left(\omega t+\alpha \right)+v_{w}\\ v_{y}=\omega r_{1}\cos\left(\omega t+\alpha \right) \end{array}$$

where v_x_ and v_y_ separately refer to X-axis speed quantity and Y-axis speed quantity.

Thus, the composite relative speed can be expressed as:

(18) $$v=\sqrt{v_{x}^{2}+v_{y}^{2}}=\sqrt{[-\omega r_{1}\sin\left(\omega t+\alpha \right)+v_{w}{]}^{2}+[\omega r_{1}\cos\left(\omega t+\alpha \right){]}^{2}}$$

The MRR of RMRP further derived by substituting Equation (12) and Equation (18) into Equation (1) as:

(19) $$\mathrm{MRR}=\frac{k}{\pi R_{1}^{2}}\int_{0}^{2\pi }d\theta \int_{0}^{R}\left\{\frac{1}{KE}\left[\mu_{0}\left(\mu_{r}-1\right)r\frac{\partial H_{r}^{2}}{\partial r}-2\rho \omega^{2}r^{2}\right]+C_{1}\right\}rdr\times \sqrt{[-\omega r_{1}\sin\left(\omega t+\alpha \right)+v_{w}{]}^{2}+[\omega r_{1}\cos\left(\omega t+\alpha \right){]}^{2}}$$

where k and C_1_ can be calculated by the experimental inversions. By measuring the MRR data of five different testing points on the workpiece (except the testing points for the experimental verification), Preston coefficient k and constant C_1_ at different testing points can be obtained. In order for it to be easier to calculate and minimize the theoretical errors, the average values of Preston coefficient k and constant C_1_, namely, modified Preston coefficient $\bar{k}$ and modified constant $\bar{C}$, are calculated.

Thus,

(20) $$\mathrm{MRR}=\frac{\bar{k}}{\pi R_{1}^{2}}\int_{0}^{2\pi }d\theta \int_{0}^{R}\left\{\frac{1}{KE}\left[\mu_{0}\left(\mu_{r}-1\right)r\frac{\partial H_{r}^{2}}{\partial r}-2\rho \omega^{2}r^{2}\right]+\bar{C}\right\}rdr\times \sqrt{[-\omega r_{1}\sin\left(\omega t+\alpha \right)+v_{w}{]}^{2}+[\omega r_{1}\cos\left(\omega t+\alpha \right){]}^{2}}$$

In the paper, the prepared MRP fluids were composed of 40 vol.% magnetic particles, 5 vol.% abrasive particles, 52 vol.% water base fluids and 3 vol.% additive. Consequently, the material parameters of MRP fluids can be considered constant. The computation parameters required to calculate Equation (20) are given in Table 1.

## 3. Simulation Analysis of the MRR Model

According to the above-mentioned analysis, when the volume content of the MRP fluids is constant, the normal polishing pressure and Preston coefficient are fixed, and the only factor that affects the MRR is the relative speed of the workpiece surface and the abrasive particles of MRP fluids. For the convenience of calculation, the angular velocity parameter ω of the workpiece is converted into the rotation speed n of the workpiece and the angular velocity parameter ω_0_ of the eccentric wheel is converted into the rotation speed n_0_ of the eccentric wheel.

In the simulation of the effect of reciprocating motion on MRR, the position parameter r of abrasive particle on the workpiece is set as from 0 to R_1_ and the initial angle α of the abrasive particle is 0. The distribution law of the material removal rate on the workpiece surface is analyzed by controlling a variable technological parameter, while the other parameters remain unchanged (* in Table). The reciprocating motion simulation parameters are selected as shown in Table 2.

### 3.1. Effect of Workpiece Rotation Speed on MRR

**Figure 5:**
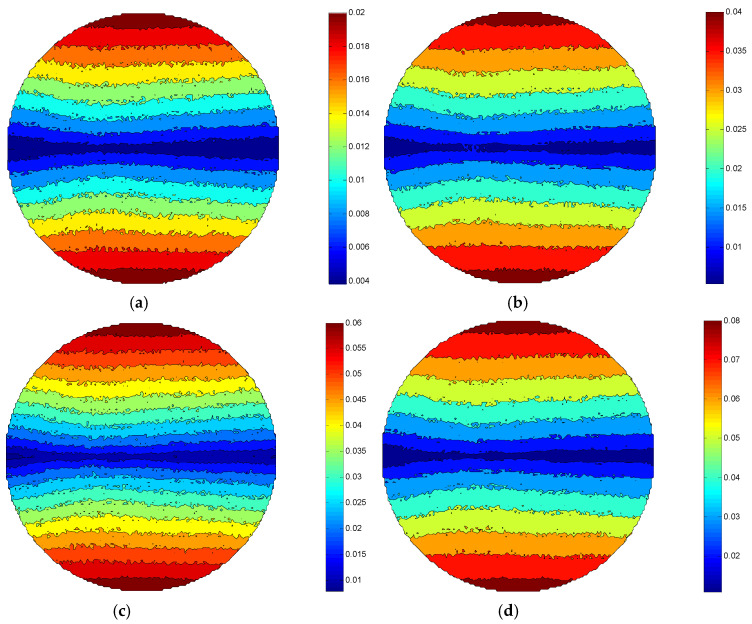
Distribution of MRR under different workpiece rotational speed conditions: (**a**) n = 150 rpm, (**b**) n = 300 rpm, (**c**) n = 450 rpm, (**d**) n = 600 rpm.

Comparing Figure 5a–d, it can be found that the MRR of the same processing area in the ±Y direction of each figure increases as the rotation speed of the workpiece is added. This is because the higher the rotational speed of the workpiece, the greater relative speed between the workpiece surface and the polishing brush. Based on the Preston equation, it is assumed that the MRR of the workpiece surface is directly proportional to the relative speed of the workpiece surface and the polishing abrasive particle under the condition of a certain Preston coefficient and polishing pressure. Therefore, the maximum material removal rate MRR_max_ of the workpiece surface in Figure 5d reaches 0.08 μm/min, while the MRR_max_ of the workpiece surface in Figure 5a is only 0.02 μm/min.

### 3.2. Effect of Eccentric Wheel Rotation Speed on MRR

**Figure 6:**
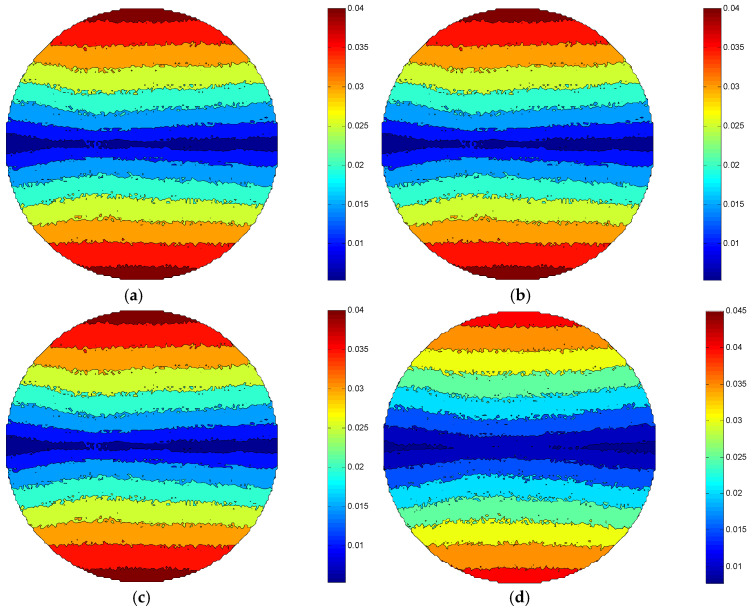
Distribution of MRR under different eccentric wheel rotational speed conditions: (**a**) n_0_ = 10 rpm, (**b**) n_0_ = 20 rpm, (**c**) n_0_ = 30 rpm, (**d**) n _0_= 45 rpm.

Comparing Figure 6a–d, it can be found that the MRR of the same processing area in the ±Y direction of each figure almost remains unchanged as the rotation speed of the eccentric wheel is added. Due to the increasing of the rotation speed of the eccentric wheel, the translation speed of the fluid carrier is enhanced, and then the combined velocity of the rotation speed of the workpiece and translation speed of the polishing brush is increased. However, compared with the rotation speed of the workpiece, the rotation speed of the eccentric wheel is far less than the workpiece’s. It can be considered that the increasing of the combined velocity of the workpiece rotation speed and the polishing brush’s translation speed can be ignored with the increasing of the rotational speed of the eccentric wheel, and the relative speed between the workpiece surface and the polishing abrasive particle is basically unchanged. Hence, under the certain conditions of the workpiece rotation speed, the MRR_max_ of the workpiece surface is all 0.04 μm/min in Figure 6a–c. Figure 6d shows that the MRR_max_ of the workpiece surface is 0.045 μm/min. This is because the relative speed between the workpiece surface and the abrasive particles is increased slightly when the reciprocating velocity of the fluid carrier reaches a certain value with the increasing of the eccentric wheel speed.

### 3.3. Effect of Eccentricity on MRR

**Figure 7:**
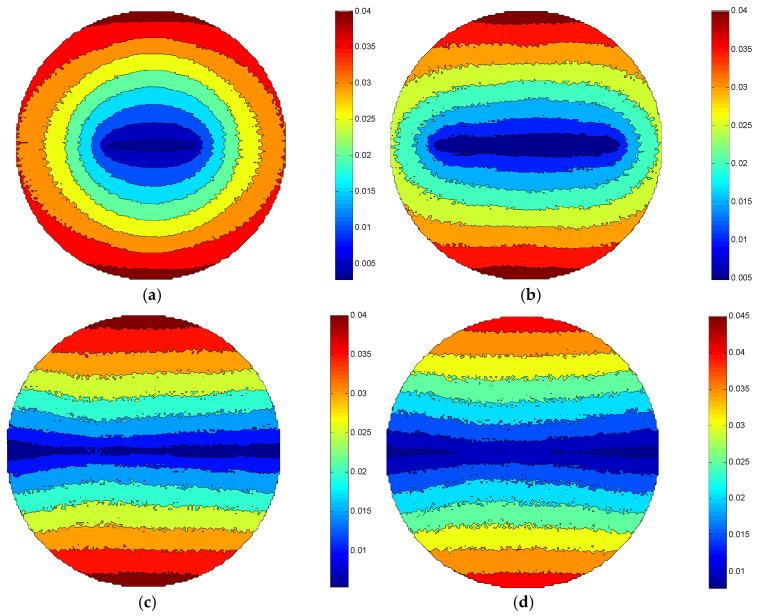
Distribution of MRR under different eccentricity conditions: (**a**) e = 0.005 m, (**b**) e = 0.01 m, (**c**) e = 0.02 m, (**d**) e = 0.04 m.

Comparing Figure 7a–d, it can be shown that the MRR of the same processing area in the ±Y direction of each figure also almost remains unchanged as the eccentricity is increased. This is because the influencing factor of the MRR is related to the relative speed of the workpiece surface and the polishing abrasive particle, while the eccentricity parameter change only affects the working stroke of the polishing brush. Nevertheless, the change of eccentricity mainly affects the MRR distribution of the workpiece, which has little effect on the MRR value in the ±Y direction of the workpiece. Therefore, under the certain conditions of the eccentricity, the MRR_max_ of the workpiece surface is also all 0.04 μm/min in Figure 7a–c. Figure 7d shows that the MRR_max_ of the workpiece surface is 0.045 μm/min. According to Equation (15), the reciprocating velocity of the fluid carrier is proportional to the eccentric wheel’s eccentricity, the relative speed between the workpiece surface and the abrasive particles is increased slightly when the reciprocating velocity of the fluid carrier also reaches a certain value with the increasing of the eccentric wheel’s eccentricity. That is why the MRR increases slightly as the eccentricity enhances.

## 4. Experimental Details

### 4.1. Preparation of the MRP Fluids

In recent years, magnetorheological fluids (MR fluids) as one of the smart materials has been attracting the attention of many scholars [33,34]. Lapping or polishing of the hard and brittle materials such as optical glass can be realized by adding a reasonable concentration of abrasive particles in MR fluids with an MR effect. However, the durability and life of MR fluids are more significant barriers to its commercial application due to actual conditions that differ from those in the laboratory [35]. Currently, most applications of MR fluids still remain in the lab testing stage.

The MRP fluids were prepared for the experiments by dispersing magnetic particles and non-magnetic abrasive particles into carrier fluids. The magnetic particles were generally required to have the characteristics of high saturation magnetization and permeability as well as low hysteresis. Among magnetic species, besides the above requirements, carbonyl iron particles (CIPs) were selected as magnetic particles of the MRP fluids because of their appropriate particle size and abundant availability. For non-magnetic abrasive particles, the cerium oxide (CeO_2_) served as abrasive particles and was widely used for polishing the K9 optical glass because of its polishing efficiency and quality. The type of carrier fluid, such as water based or oil based, was a key factor for the MRP fluids, and some studies have paid attention to the base medium of the MRP fluids. To obtain higher polishing efficiency for the K9 optical glass, deionized water as water-based carrier fluids was chosen as base medium of the MRP fluids. That is because deionized water has a certain chemical etching effect on the K9 optical glass, which helps the optical glass processing. Water soluble sodium dodecyl sulfonate (SDS) served as stabilizing additives was added to the MRP fluids to prevent the sedimentation of the CIPs. In the experiments, on the one hand the MRP fluids could be fully mixed during the RMRP; on the other hand, shear rate, temperature and duration were within the appropriate index range. Consequently, the service life of the MRP fluids could not be considered. The specific parameters of the MRP fluids are shown in Table 3.

### 4.2. Experimental Conditions and Measurement Methods

To verify the correctness of the MRR model, the experimental setup of RMRP was used to machining the optical glass as shown in Figure 2. By applying the Croma10218 coordinate measuring machine (Shenzhen, China), the Axio Imager A2m microscope (Zeiss, Oberkochen, Germany) and OLS 4100 laser scanning confocal microscope (Tokyo, Japan), the MRR of RMRP was measured and the surface morphology of the workpiece was observed. The K9 optical flat glass (Shenyang, China) with the initial surface roughness of Ra 320–330 nm and the radius of 15 mm was adopt for the experimental workpiece. Other experimental parameters were an electromagnet coil current of 3 A, a working gap of 1 mm and a processing time of 45 min.

**Figure 8:**
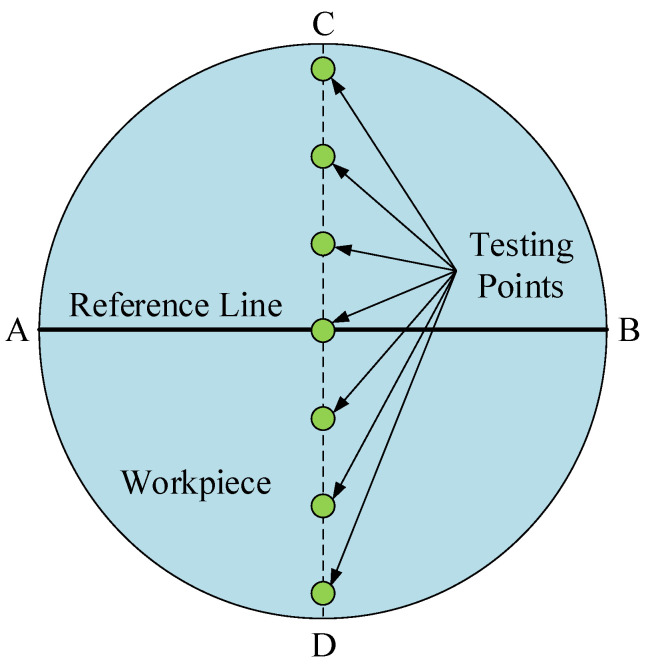
The schematic diagram of selected testing positions.

A reasonable measurement of the MRR is the key to verify the MRR model. In this study, the diameter of the workpiece used as the reference line (AB line) was required to be parallel to the translation direction of the fluid carrier when the workpiece was installed before the RMRP. Moreover, 7 equidistant points from the vertical line of the reference line (CD line) on the workpiece were chosen as testing points, and the average MRR of 7 testing points was selected as the measurement result, as shown in Figure 8. The selection of experimental parameters was the same as the selection of simulation parameters, as shown in Table 2, and the change of the MRR was also studied by controlling the selection principle of a parameter change. In the meantime, in order to ensure the accuracy of the measurement results and reduce experimental errors, the average value of 5 measurements at a certain testing point was taken as the measurement result of the MRR at that point.

## 5. Results and Discussion

### 5.1. Effect of Workpiece Rotation Speed on MRR

**Figure 9:**
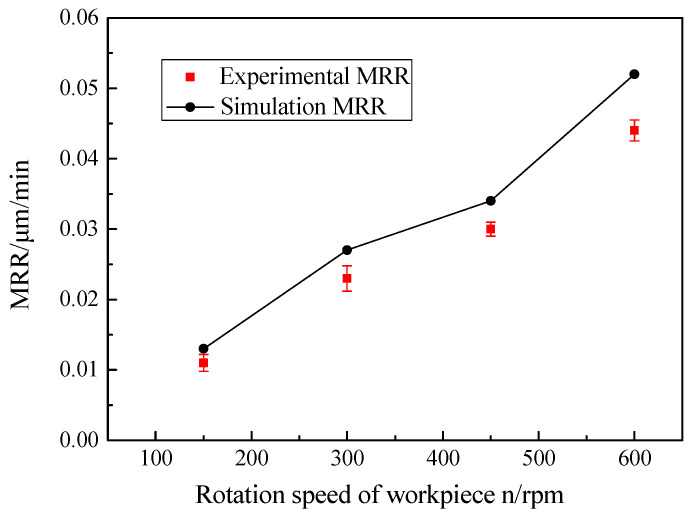
Effect of workpiece rotation speed on MRR.

As shown in Figure 9, with increasing the rotation speed of the workpiece, the MRR grew significantly. The reason for this was that when the rotational speed of the workpiece increased, the speed of the same testing points on the workpiece rose, and then the relative speed of the workpiece surface and the polishing abrasive particle enhanced. Thus, based on the Preston equation, the experimental results of the MRR showed an increasing trend which was consistent with the theoretical values when the Preston coefficient and polishing pressure were fixed. As the rotational speed of the workpiece increased from 150 to 600 rpm, the experimental MRR values added from 0.0115 ± 0.0012 to 0.0443 ± 0.0015 μm/min. The average relative error between the theoretical and experimental values was 16.77%, and it is proved that the MRR model of RMRP was effective. Compared with the theoretical values, the reason why the experimental MRR values of the workpiece were slightly lower may be that the polishing abrasive particles in the machining process were affected by the rotational centrifugal force of the workpiece, and the number of effective polishing abrasive particles on the workpiece surface was reduced.

**Figure 10:**
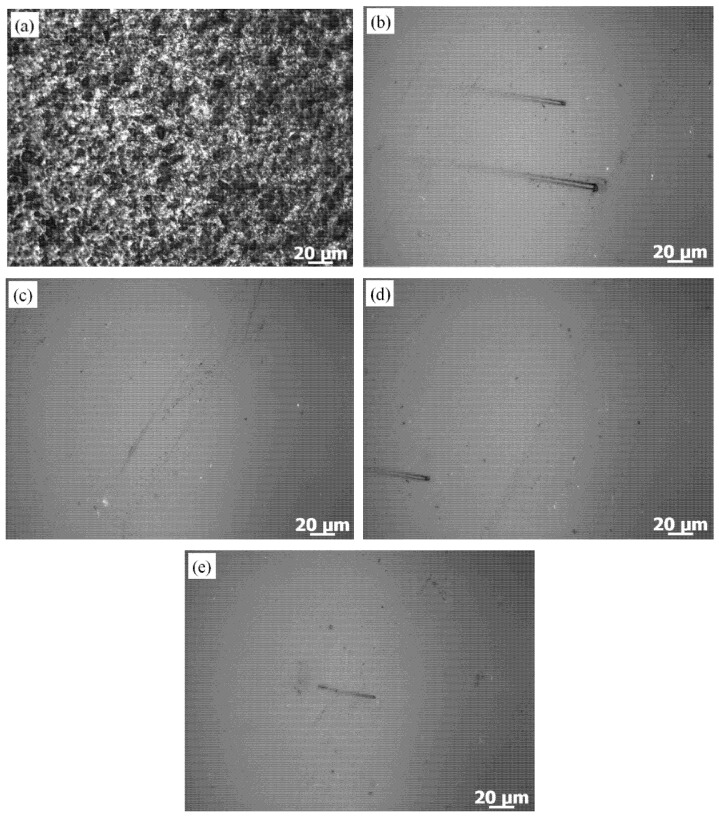
The surface morphologies with different rotational speeds of the workpiece: (**a**) initial surface morphology, (**b**) n = 150 rpm, (**c**) n = 300 rpm, (**d**) n = 450 rpm, (**e**) n = 600 rpm.

The surface morphologies with different rotational speeds of the workpiece are shown in Figure 10. It can be found that there was obvious improvement in the workpiece surface morphologies as compared to the initial surface morphology. The microscopic convex peaks on the workpiece surface had been removed on a large scale, and the workpiece surface became smoother as the rotational speed of the workpiece increased. The polished workpiece surface roughness reduced to Ra 50.8 ± 1.2 from initial Ra 330.3 ± 1.6 nm by using a TR3230 surface roughometer (Beijing, China) when the technical parameters of the workpiece rotation speed of 300 rpm, the eccentric wheel rotation speed of 20 rpm and the eccentricity of 0.02 m were applied.

### 5.2. Effect of Eccentric Wheel Rotation Speed on MRR

**Figure 11:**
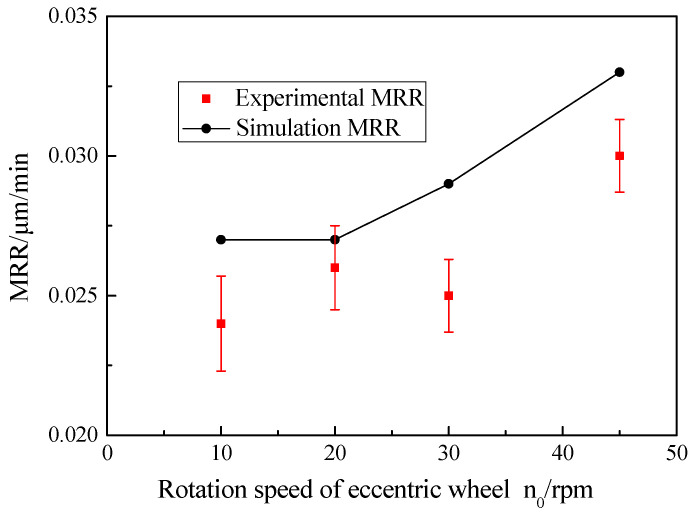
Effect of eccentric wheel rotation speed on MRR.

As shown in Figure 11, with increasing the rotation speed of the eccentric wheel, the experimental MRR values of the workpiece changed slightly, which had little effect on the MRR of the workpiece. The reason for this was that the main purpose of the reciprocating movement of the magnetic polishing brush was to equalize the polishing force on each area of the workpiece, whereas the contribution to the material removal of the workpiece was not large. Consequently, the experimental MRR values of the workpiece had little change, ranging from 0.0242 ± 0.0017 to 0.031 ± 0.0013 μm/min, and the average relative error between the theoretical and experimental values was 10.59%. Compared with the theoretical values, the experimental MRR values of the workpiece also showed that the experimental results were slightly lower.

**Figure 12:**
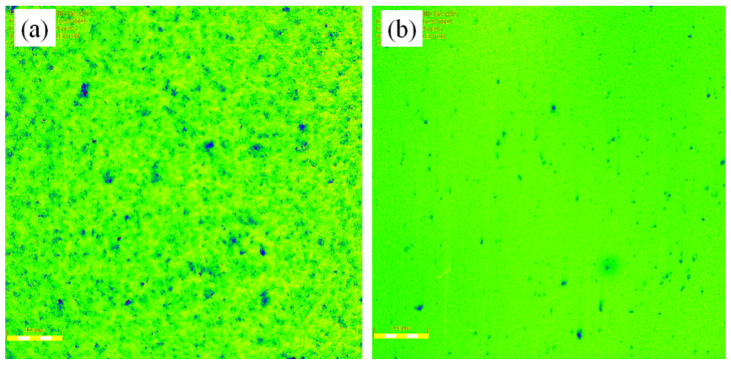
The morphology images of the polished workpiece surface: (**a**) before and (**b**) after being polished.

Figure 12 showed the morphology images of the polished workpiece surface by using the OLS 4100 laser scanning confocal microscope that can synthesize three-dimensional images for non-metallic material. It was found that the original microscopic convex peaks on the workpiece surface were worn away, obtaining the desired polishing effectiveness. The polished workpiece surface roughness reduced to Ra 60.4 ± 1.6 from initial Ra 321.0 ± 1.2 nm when the technical parameters of the workpiece rotation speed of 300 rpm, the eccentric wheel rotation speed of 45 rpm and the eccentricity of 0.02 m were applied.

### 5.3. Effect of Eccentricity on MRR

**Figure 13:**
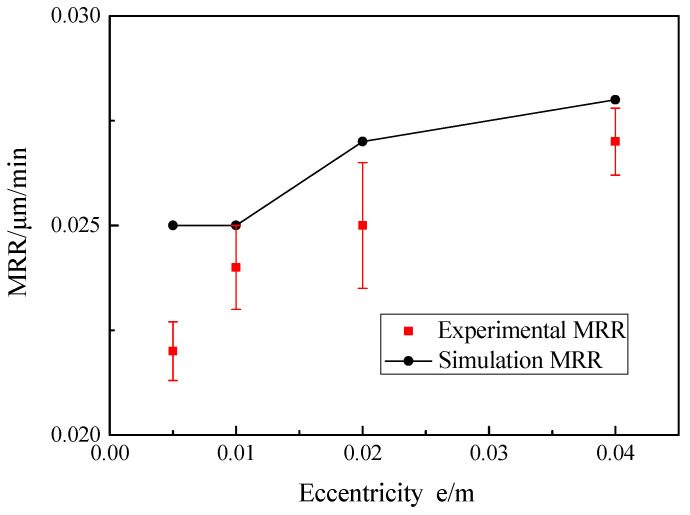
Effect of eccentricity on MRR.

The eccentricity had little effect on the material removal of the workpiece surface—that is, the experimental and theoretical MRR values of the workpiece at the testing point are slightly changed. The experimental MRR values were obtained between 0.0220 ± 0.0007 and 0.0273 ± 0.0008 μm/min by applying the Croma10218 coordinate measuring machine as shown in Figure 13, and the average relative error between the theoretical and experimental values was 7.38%. The reason for this was that with the increasing eccentricity, the relative speed between the workpiece surface and the abrasive particles changed slightly, then the change of material removal rate was not obvious. Hence, the results indicate that eccentricity had little effect on the MRR through the measurement of the testing points.

## 6. Conclusions

Based on the RMRP principle and Preston equation, the MRR model of the RMRP as well as its normal polishing pressure model was established in this study. On this basis, the effects of different technological parameters on the MRR of the workpiece were investigated theoretically and experimentally. The following conclusions were drawn:

1. The K9 optical flat glass was polished with the RMRP setup to study the effects of technological parameters on the MRR of a workpiece. The experimental results were in good agreement with the theoretical results under the same technical parameters and the average relative errors between the theoretical and experimental values were 16.77%, 10.59% and 7.38%, respectively, according to the effects of workpiece rotation speed, eccentric wheel rotation speed and eccentricity on MRR, then the efficacy of the MRR model of the RMRP was verified.
2. It was found that the surface roughness reduced to Ra 50.8 ± 1.2 from initial Ra 330.3 ± 1.6 nm when the technical parameters of the workpiece rotation speed of 300 rpm, the eccentric wheel rotation speed of 20 rpm and the eccentricity of 0.02 m were applied; the surface roughness reduced to Ra 60.4 ± 1.6 from initial Ra 321.0 ± 1.2 nm when the technical parameters of the workpiece rotation speed of 300 rpm, the eccentric wheel rotation speed of 45 rpm and the eccentricity of 0.02 m were applied.
3. As the rotational speed of the workpiece rose, the MRR of the workpiece increased significantly, and the experimental MRR values added from 0.0115 ± 0.0012 to 0.0443 ± 0.0015 μm/min. Compared with the effect of workpiece rotation speed on the MRR, the effect of eccentric wheel rotation speed and eccentricity on the MRR could be neglected.

## Figures and Tables

**Table 1:** The computation parameters of material removal rate (MRR) by the reciprocating magnetorheological polishing (RMRP).

Computation Parameters	Values
Permeability of vacuum $\mu_{0}$/H/m	$4\pi \times 10^{-7}$
Relative permeability of MRP fluids $\mu_{r}$	3
Material characteristics of MRP fluids KE	2.9
Density of MRP fluids $\rho /\mathrm{kg}/m^{3}$	1980
Radius of the polishing brush R/m	0.015
Radius of the workpiece R_1_/m	0.015
Modified Preston coefficient $\bar{k}$/m^2^/N	$6.7\times 10^{-12}$
Modified constant $\bar{C}$	37.8
Working gap h/m	0.001

**Table 2:** Simulation parameters of reciprocating motion.

Simulation Parameters	Values			
Rotation speed of workpiece n/rpm	150	300 *	450	600
Rotation speed of eccentric wheel n_0_/rpm	10	20 *	30	45
Eccentricity e/m	0.005	0.01	0.02 *	0.04

* Invariable technological parameter value.

**Table 3:** Proportion and particles size of the MRP fluids.

Constituents of MRP Fluids	Volume Fraction (vol.%)	Size (μm)
CIPs	40	2.2
CeO_2_	5	2.5
SDS	3	-
Deionized water	52	-
